# Supplementary material for: Are carbon emissions trading and green financial instruments synergistic? -Comprehensive quantitative research based on content analysis
Source: PLoS One. 2024 Mar 7;19(3):e0298601. doi: 10.1371/journal.pone.0298601 (PMC10919601; doi:10.1371/journal.pone.0298601)
Supplement: S1 Data — (ZIP) [file pone.0298601.s001.zip › Data/地级市Z_F工作报告.zip!/╣π╬≈/╖└│╟╕█╩╨/└┤╫╘╣1⁄2╓┌║┼í╛┬φ┐╦╩2╛▌═°í┐_2007.docx]

各位代表：

　　现在，我代表市人民政府向大会作政府工作报告，请予审议，并请市政协各位委员和其他列席人员提出意见。

率先发展的基础更加坚实

　　2006年是我市迈出率先发展步伐、各项工作取得新进展的一年。在自治区党委、自治区人民政府和市委的正确领导下，在市人大、市政协的监督支持下，全市各族人民高举邓小平理论和“三个代表”重要思想伟大旗帜，全面落实科学发展观，紧紧围绕“三个率先”和经济发展“双跨越”目标，抢抓广西北部湾经济区开放开发及多区域合作新兴机遇，克难攻坚，乘势而上、乘势而为，较好地完成了市三届人大七次会议确定的目标任务，实现了“十一五”的良好开局。

　　经济快速增长，“双跨越”目标顺利实现。初步核算，生产总值突破100亿元,达115.69亿元，增长17.1％，增幅全区第二；财政收入突破10亿元，达10.59亿元，增长31.7％，增幅全区第一。经济发展“双跨越”目标顺利实现，经济发展呈现建市以来速度最快、效益最好、景气指数最高的态势。

　　港口发展提速，货物吞吐量突破3000万吨。全市港口货物吞吐量完成3382万吨，增长42.3％，其中：港务集团2506万吨，增长24.9％。新开通两条集装箱班轮航线，集装箱完成13.03万标准箱，增长24.1％。20万吨级泊位及其配套航道试产，13－14＃泊位和5万吨级液体化工码头相继建成投产。

　　投资增势强劲，发展后劲进一步增强。全社会固定资产投资68.96亿元，增长60.5％，增幅全区第一，对经济增长的贡献率超过80％。沿海基础设施建设大会战一期10个项目基本完成，二期8个实施项目全部开工建设。重大项目建设加快，防城港电厂累计完成投资30多亿元，惠禹饲料蛋白项目建设加快，防城港钢铁基地通过专家组评估，广西白龙核电站一期工程筹建处正式挂牌成立。城镇建设投资首次突破10亿元；市四家班子顺利搬迁行政中心区，“还岛于港”战略迈出关键一步；房地产业投资5.42亿元，增长108.2％，增幅全区第一。

　　产业结构优化，工业经济主导局面初步形成。三次产业结构由2005年的23.9:38.0:38.1调整为21.9:41.7:36.4，二产的比重超过三产。规模以上工业总产值114.61亿元，增长32.4％；规模以上工业增加值28.55亿元，增长34.3％；规模以上工业盈亏相抵实现利润7.01亿元，增长1.74倍；企业技改投资完成5.95亿元，增长90.7％；规模以上工业对经济增长的贡献率为42.1％，拉动经济增长7.2个百分点。

　　“三农”工作加强，县域经济全面发展。农业总产值42.46亿元，农村经济总收入51.51亿元，分别增长7.9％和5.8％；2005/2006榨季全市糖料蔗总产量突破200万吨，增长34.7％；农村基础设施逐步改善，投入6000多万元建设农村公路、乡镇客运站和便民码头；农业产业化进程加快，农业产业化经营组织突破100个。县域经济快速发展，区、县（市）生产总值82.69亿元，增长16.9％，占全市生产总值的71.5％。

　　开放合作扩大，外贸外资外经工作取得新成效。外贸进出口总额10.29亿美元，增长21.1％；直接利用外资4967万美元，增长24.9％；新引进投资项目55个，总投资48.51亿元，新增招商引资到位资金23.80亿元。成功举办2006中越边境（东兴—芒街）商贸·旅游博览会，签订项目合同、协议24个，投资总额11.12亿元，商品成交额7200万元。

　　人民生活水平提高，和谐社会建设扎实推进。农民人均纯收入3172元，新增468元，增长17.3％，是建市以来增加最多、增幅最大的一年；城镇居民人均可支配收入9113元，增长25.6％，增幅全区第一。社会消费品零售总额26.05亿元，增长14.5％。金融机构本外币存贷款余额分别为98.23亿元和58.15亿元，分别增长21.8％和34.2％，贷款余额增幅全区第一。就业再就业和社会保障工作取得新成效，新增城镇就业9229人，再就业1390人，城镇登记失业率控制在4.0％之内。整村推进扶贫开发工作成效明显，投入2532万元加强贫困村的基础设施建设，解决6500多农村贫困人口温饱问题和6.15万农村人口饮水难问题。科技工作不断进步，实施科技项目47项，引进推广农业新品种、新技术50项，培训农民9.5万人次。教育事业取得新发展，“两基”成果得到巩固，“两免一补”落到实处，高考上线率82.3％，创历史新高。文化工作取得新成绩，获自治区级以上文化奖项20个，白龙古炮台被列入国家重点文物保护单位，填补了我市该项空白。卫生工作得到加强，全市实现了乡镇卫生院以上医疗机构突发公共卫生事件应急网络直报，新型农村合作医疗参合率达62.7％。广播电视事业有新发展，有线电视数字化整体转换顺利完成。城乡清洁工程取得初步成效。人口计生、国土、环保、统计、民政、外事、民族、宗教、侨务、体育等各项工作进一步加强。工青妇的作用进一步发挥。精神文明建设和平安防城港建设力度加大。安全生产监督管理工作上新水平，安全生产事故下降7.2％，死亡人数下降11.0％。社会治安综合治理得到加强，“打黑除恶”等专项斗争取得实质性成效。整顿市场秩序和打私工作有效开展。双拥工作扎实推进，军政、军民关系更加密切。边境稳定，社会和谐，人民安居乐业。

　　政府职能转变，民主法制建设不断加强。政府执行力和公信力明显提升，效能投诉比上年下降19.4％。全市受理效能投诉331件，办结311件，办结率94.0％，追究了13名机关工作人员效能责任。坚持向市人大及其常委会报告工作制度，自觉接受人大监督，全面落实有关决议、决定，办理人大代表建议、批评和意见52件，办复率100％。加强与市政协及各民主党派的联系和沟通，主动接受民主监督，办理政协委员提案126件，办复率100％。普法教育深入开展，依法行政继续推进，政府廉政建设和反腐败工作取得新成效。

　　回顾过去的一年，我们深深体会到：只有坚持以科学发展观统领全局，抢抓新兴机遇，狠抓项目投资，加强经济运行监控，促进社会各项事业全面进步，才能加快发展、率先发展，最终实现富裕和谐防城港的奋斗目标。

　　各位代表！

　　过去一年，我市经济社会发展是在克服建设资金紧缺、天气持续干旱等诸多困难的情况下取得的，成绩来之不易。这是贯彻落实党的十六届五中、六中全会精神和自治区党委、自治区人民政府及市委正确决策的结果，是市人大、市政协有效监督和大力支持的结果，是中直、区直单位和驻我市部队支持帮助的结果，是全市各族人民团结奋斗的结果。在此，我谨代表市人民政府，向全市广大干部群众，向解放军指战员、武警官兵和公安民警，向各民主党派、工商联、无党派人士、人民团体，向所有关心、支持、参与我市现代化建设的各界人士和海内外朋友，表示衷心的感谢和崇高的敬意！

　　在肯定成绩的同时，我们也必须清醒地认识到，在前进的道路上还面临着不少困难和问题：经济总量小，结构不合理，增长方式有待转变；建设资金筹措难度大，基础设施不够完善，工业化、城镇化水平不高；人才资源匮乏，企业自主创新能力不强；机关效能和服务水平有待提高；政府性债务沉重等。对于这些困难和问题，在今后的工作中，我们要采取更加有力的措施，妥善加以解决。

2007年的总体要求和主要目标

　　今年是我市打牢基础、奋力爬坡、率先发展的关键一年。政府工作的总体要求是：以邓小平理论和“三个代表”重要思想为指导，认真贯彻党的十六大、自治区九次党代会和市四次党代会精神以及全区、全市经济工作会议精神，全面落实科学发展观，抢抓多区域合作新兴机遇，以推进工业化、城镇化为主导，以加快重大项目建设、扩大经济总量为重点，以开放合作、改革创新为动力，扎实推进经济建设、政治建设、和谐社会建设，着力保障民生、改善民生，促进经济社会又好又快发展。

　　经济社会发展主要目标是：生产总值增长17％；财政收入增长20％，其中：一般预算收入增长15％；全社会固定资产投资增长32％；工业总产值增长28％，其中：规模以上工业总产值增长35％；港口货物吞吐量增长25％，其中：港务集团增长20％；社会消费品零售总额增长15％；外贸进出口总额（海关）增长17％；外商直接投资增长37％；城镇居民人均可支配收入增长10％；农民人均纯收入增长10％；城镇新增就业9000人，人口自然增长率控制在11‰以内；万元生产总值能耗下降1.6％，万元规模以上工业增加值能耗下降1.6％。

　　在今年经济社会发展目标中，节能降耗、人口增长两个指标是约束性的，要想方设法完成。生产总值、财政收入、全社会固定资产投资、工业总产值、港口货物吞吐量等指标是预期性的、指导性的，在实际工作中，我们将根据经济社会发展情况适时进行调整，提出新的、更高的要求。

　　做好今年的工作，我们有不少有利条件。一是机遇难得。加快广西北部湾经济区开放开发，推动泛北部湾合作得到了中央肯定，中央领导要求广西沿海发展应形成新的一极；全国政协把广西北部湾经济区开放开发列为今年专题协商议题；《国家西部大开发“十一五”规划纲要》把广西北部湾经济区列入三个率先发展的重点区域之一。我市作为广西北部湾经济区的核心区域，占有加快发展、率先发展的各种先机。二是态势良好。在“十五”快速发展的基础上，全市经济增长提速、效益趋好、实力增强，“三个率先”起步良好，产业发展更加协调，改革开放更加深化，人民生活更加富裕，经济社会发展更加和谐。三是后劲增强。防城港钢铁基地、广西白龙核电站等一批重大项目进展顺利，中油能源、惠禹饲料蛋白、防城港电厂、宏源浆纸、上上糖业、昌菱糖厂等重点企业和骨干企业今年将投产、达产或扩产，基础设施建设全面铺开，重点推进，这对经济增长是一个强有力的支撑。

　　要实现今年经济社会发展目标，必须努力做到：

　　——坚持科学发展。坚持以科学发展观统领经济社会发展全局，统筹推进工业化、城镇化和社会主义新农村建设，努力实现速度、质量、效益相协调，消费、投资、出口相协调，人口、资源、环境相协调，不断开创科学发展新局面。

　　——坚持开放发展。切实把握千载难逢的多区域合作新兴机遇，深入实施互利共赢的开放战略，加快对外开放步伐，加大招商引资力度，主动承接产业转移；切实转变工作作风，加强机关行政效能建设，优化发展环境。以环境促开放，以开放促开发，以开发促发展，努力把我市打造成为泛北部湾经济发展新高地。

　　——坚持务实发展。大兴求真务实之风，牢固树立“清简务本、行必责实”的良好作风，把工作的着力点真正放到研究解决改革发展稳定中的重大问题上，放到研究解决群众生产生活中的紧迫问题上，放到研究解决经济建设中的突出问题上，坚持以严谨细致的作风去抓落实、求实效、促发展。

　　——坚持和谐发展。始终高度关注民生，把实现好、维护好、发展好人民群众的根本利益作为一切工作的出发点和落脚点。通过发展经济，加大公共服务投入，着力改善民生，努力为民办实事，不断提高人民群众的生活水平，让全市人民共享改革发展的成果。

保持经济社会又好又快发展

为促进我市经济社会又好又快发展，今年必须重点抓好以下十项工作：

一、加快港口建设与发展，提升港口核心竞争力

　　加强港口基础建设。按照大型化、专业化要求，加快基础设施建设。力争开工建设防城港钢铁基地20万吨级矿石码头及陆域形成工程，防城港18－22＃泊位、501－502＃泊位；大力发展集装箱专用码头，加快配套铁路、航道、库场及其他服务设施。

　　创新港口经营模式。加快实施“以港引工、以工促港、港工互动”发展战略，形成前港后厂、前港后区、前港后园的格局；抓住广西北部湾国际港务集团成立的机遇,整合港口资源，创新经营模式，提升港口核心竞争力。进一步加强企沙、白龙、茅岭、潭油、潭吉、竹山等中小港口的规划、建设和管理，形成全市港口发展“一盘棋”的局面。

增强港口疏运集散能力。充分利用“防港物流”品牌效应，加强与国内外物流企业的合作，大力发展现代物流业。发挥路网优势，增加海运航线，打造四通八达、快捷方便的物流网络；大力发展物流企业，加快物流园区建设。

二、扎实推进工业化，进一步强化工业主导地位

　　建成投产一批重大工业项目。大力支持大海粮油增资扩建、益海大豆、农垦防城分公司精制糖等项目的建设，惠禹饲料蛋白和万鑫钢铁二期项目要尽快建成投产；加快推进防城港电厂建设，确保按计划投产发电，并抓紧二期项目建设的前期工作；加快天宝300万吨重交沥青项目工程进度。

　　加快推进工业园区建设。按照“总体规划、分期建设”的原则，大力推进企沙工业区10－15平方公里核心区、东兴江平工业园区的建设，努力将其打造成现代制造业的集中区、吸引外资的聚集区、循环经济的示范区；继续抓好港口渔洲城和公车、防城河西和茅岭、上思城西等县域工业园区的规划建设。

　　大力发展循环经济。围绕“十一五”期末万元生产总值能耗比2005年降低8％的目标，突出抓好创建环境友好型企业和循环经济、清洁生产的试点工作。加快建设市污水处理厂和市垃圾无害处理二期工程，加快推进东兴、防城、上思污水处理和垃圾处理项目；扩大排污许可证制度的实施范围，实行污染物排放总量和浓度的双向控制。

三、加快重大项目建设，增强投资拉动力

　　推进重大项目建设。尽快完成沿海基础设施建设大会战一期项目，完成二期大会战项目总投资的40％，全部项目力争在2008年自治区成立50周年大庆前竣工投产。如期完成防城港电厂、大海粮油三期等重大项目的投资，积极推进防城港钢铁基地、广西白龙核电项目的各项工作，加快防城南至企沙铁路支线、崇左至钦州高速公路上思段、江山半岛旅游景区道路、江山至东兴一级公路后续工程、江山至白龙二级公路等交通基础设施建设，做好中越北仑河二桥、钢铁基地配套公路等项目前期工作。

　　多渠道筹措建设资金。健全重点项目库，积极向国家开发银行、世行、亚行和其他商业银行争取贷款，向北部湾开发投融资平台争取项目资金。合理利用现有土地，盘活土地资源，为项目建设提供资金支持。通过独资、合资或采用出让、租赁、抵押等方式，筹集建设资金，形成项目建设投资多元化格局。

　　加强项目建设管理。强化项目建设目标责任制，完善市领导挂钩联系、职能部门具体负责、相关部门积极配合的项目建设机制。严格落实项目法人责任制、招投标制、施工监理制和工程审计制，建立健全投资决策机制和投资监管体系。抓好重大项目审计，加强检查监督，严格资金管理与工程监理。

四、加强城镇规划管理，全力推进城镇化进程

　　强化城镇群整体规划。按照广西北部湾经济区开放开发的要求，围绕建设临海临港工业基地、西南出海国际大通道和打造物流新高地的目标，坚持走新型城镇化道路，完善编制、加快实施各城镇总体规划和土地利用、主体功能区建设等专项规划。推进乡镇和重点村规划。

　　完善城镇发展格局。加快城市中心区开发，打造核心城区，辐射带动港口、防城城区发展，完善城市要素体系，优化城镇空间布局；加快东兴、上思城区建设；优先发展企沙、那良、江平、在妙等中心镇，兼顾发展其他小城镇，加快培育形成沿海城镇带、边境城镇带、中心城镇群、山区城镇群等“两带两群”的城镇发展格局。

　　深入实施城乡清洁工程。加大市容市貌综合整治力度，搞好城市绿化、亮化、净化和美化，把市容市貌综合整治纳入各级政府的重要考核内容，严格考核，严格责任追究。创新城市管理思路，理顺部门职能，整合城市管理资源，充分发挥城区、街道和社区在城市管理中的作用，形成市区衔接、行为规范、调控有力的长效管理机制。

五、加大“三农”工作力度，扎实推进新农村建设

　　发展现代农业。用现代科学技术改造农业，用现代产业体系提升农业，用现代经营形式推进农业，用现代发展理念引领农业，培育新型农民发展农业。提高农业水利化、机械化和信息化水平；提高土地产出率、资源利用率和劳动生产率；提高农民素质，提高农业效益和竞争力。

　　壮大县域经济。采取市场引导、政策扶持、信息服务和加大资金投入、加强技术指导等措施，以海产品加工、制糖制药、竹木浆纸、八角肉桂和金花茶、火龙果加工为重点，培育一批规模大、效益好、辐射带动力强的加工龙头企业，形成县域特色产业集群，以县域工业辐射带动县域经济发展。

　　推进新农村建设。加快农田水利等农村基础设施和公共事业建设，年内实现所有乡镇通油路或水泥路；突出抓好防汛防台风和抗旱减灾体系建设；进一步加强农村生态能源建设，改善农村生产生活条件。推进农村综合改革；认真落实解决农民工问题各项政策措施；继续做好重大动物疫病的防治和大中型水库移民后期扶持政策实施工作。

六、加大招商引资力度，努力实现开放合作新突破

　　加快引进一批重大项目。充分利用中国—东盟博览会和中国—东盟商务与投资峰会以及中越边境（东兴—芒街）商贸·旅游博览会等平台，广泛进行网络招商、品牌招商、委托招商、代理招商、以商招商、上门招商等，有针对性地引进一批项目，力争与世界500强、大型央企合作取得重大突破。

　　注重招商引资实效。依托项目库有针对性地提供产业和项目信息，有针对性地招大商，进一步提高招商引资水平。切实提高项目履约率、开工率和投产率；抓紧跟踪落实意向项目，力争尽快签约落户。

　　努力扩大对外贸易。重点扩大对东盟，特别是对越南贸易进出口，转变外贸增长方式，优化出口商品结构，提高产品质量。大力发展边境贸易。加快实施电子口岸和“大通关”工程。

七、加强财税金融工作，为经济发展提供有力支撑

　　狠抓财源培育。重点抓重大项目、重点企业、重点行业的税源培育，做大做强支柱财源。强化征管，依法征税，努力做到应收尽收，应免尽免；加强非税收入管理，严格执行收支两条线。建立健全收入增长激励机制，充分调动征管部门和各区、县（市）的积极性。

　　争取上级财政支持。认真研究一般性转移支付、专项转移支付、“三奖一补”以及各种专项资金的扶持方向和财政政策，争取自治区财政和有关部门更多的资金支持。

　　加强财政支出管理。深化部门预算、政府采购、国库集中支付制度改革。坚持量力而行、量入为出，厉行节约，勤俭办事业，把有限的资金真正用在刀刃上。强化预算约束，严格控制零星追加；优化支出结构，提高资金使用效率。

　　发展金融业务。支持金融事业发展，强化金融服务，努力改善金融生态环境；加快中小企业信用担保体系建设，积极搭建银企合作平台，建立互信共赢的银企关系；稳步推进农村信用社改革，加大不良贷款清收力度；鼓励金融机构主动融入广西北部湾经济区开放开发，扩大贷款规模，实现经济、金融良性循环。

八、促进民营经济和服务业发展，开创全民创业新局面

　　壮大民营经济。落实和完善加快民营经济发展的相关政策措施，营造公平竞争的市场环境和一视同仁的政策环境，进一步破除制约民营经济发展的体制性障碍，努力解决民营企业市场准入门槛高、融资难等问题。支持和帮助有条件的民营企业壮大规模、提升层次，引导民营企业理性投资、健康发展。

　　发展现代服务业。做大做强旅游产业，加快京岛风景名胜区、十万大山国家森林公园等创4A建设步伐，推进东兴市争创全国优秀旅游城市；拓宽旅游产业融资渠道，鼓励和吸纳社会资本投资兴办旅游业。积极发展消费信贷，培育住房、汽车等消费热点，促进消费结构升级。积极发展法律、会计、评估、社区服务等新兴服务业。

九、大力发展各项社会事业，促进社会和谐

　　随着财政收入的不断增长，要继续调整和优化财政支出结构，加大对公共社会事业的投入，让全市各族人民共享改革发展成果，不断促进社会和谐。

　　做好人口和计划生育工作。千方百计稳定低生育水平，努力遏制出生人口性别比偏高的势头，不断完善流动人口管理服务体系，落实计划生育奖励优惠政策，严格执行“一票否决”制度，确保完成年度目标管理责任制。

　　优先发展教育。全面实施素质教育，优化教育资源配置，整合公共教育资源，推动城乡教育协调发展。巩固发展“两基”工作成果，迎接国家评估验收；积极创造条件，做好市职业技术学院建设前期工作。

　　深入实施人才强市战略。抓住人才培养、吸引、用好三个环节，建立完善激励优秀人才脱颖而出、人尽其才的有效机制和良好环境，为经济社会发展提供支撑和保障。

　　抓好科技工作。实施科技发展中长期规划纲要和第三轮创新计划，完善自主创新的体制机制，落实鼓励创新的相关政策，推进创新科技服务网建设，进一步提高科技对经济增长的贡献率。

　　加强公共卫生体系建设。加大卫生资源整合力度，加强公共卫生、农村卫生和城市社区卫生建设，切实做好重大传染病防控，提高公共卫生服务和应急救治能力，保障人民健康。

　　发展和谐文化。牢固树立社会主义荣辱观，积极发展文学艺术、新闻出版和广播影视业，实施新农村文化致富工程。加强文化、体育基础设施建设，力争开工建设防城港市图书馆，加快东兴民族生态博物馆建设。广泛开展全民健身活动，大力倡导文明健康生活方式。

　　建设平安防城港。牢固树立“安全发展”理念，不断提高安全生产监督管理水平；加强社会治安综合治理，维护社会稳定；推进“五五”普法教育；促进民族团结进步；深入开展拥军优属、军民共建、爱民固边活动，巩固和发展军政军民团结，再创全国双拥模范城。

十、高度重视民生，努力为民办好八件实事

　　以解决人民群众最关心、最直接、最现实的利益问题为重点，集中力量办好八件实事。

　　（一）着力改善华侨农林场的生产生活条件。多渠道筹措专项建设资金600万元（包括中央、自治区和地方配套资金），重点改善十万山等特困华侨农林场基础设施，解决群众行路难、上学难、看病难、饮水难、住房难等问题。

　　（二）加快兴村富民整村推进。加大扶贫工作力度，投入1150万元，加强贫困村基础设施建设；实现农村实用技术培训3000人、帮扶脱贫7000人的目标。

　　（三）全面推进新型农村合作医疗建设。新型农村合作医疗参合率达到80％以上，缓解农民看病难、看病贵的问题。

　　（四）实施城镇居民安居工程。用足用活住房公积金政策，在自治区政策许可范围内逐步提高公积金缴存比例；着力推进市公务员小区建设；实行城市廉租住房制度，缓解部分低收入家庭住房难问题。

　　（五）实施城乡就业工程。实现城镇新增就业人员9000人，再就业1350人，城镇登记失业率控制在4.0％以内；新增农村劳动力转移就业1万人。

　　（六）加大社会保险工作力度。企业职工养老保险参保人数达到3.8万人，失业保险参保人数达到4.4万人，医疗保险参保人数达到5.5万人。

　　（七）设立助学帮困基金。加大对贫困家庭子女教育的资助力度，重点资助考上大学的贫困家庭子女，确保考上大学的贫困学生都能上大学，让所有孩子都能上得起学。

　　（八）设立艾滋病防治基金。在全力争取中央、自治区资金的同时，逐步加大投入，切实做好艾滋病防治工作。

十一、大力推进政府自身建设

　　加强机关行政效能建设。贯彻实施行政许可法，深化行政审批制度改革；有针对性地解决影响投资环境、损害政府形象、制约经济发展等问题。建立健全提高行政效能的长效机制，进一步健全首问负责制，全面推行限时办结制，严格实行责任追究制，推行重大事项专人负责制，在全市集中开展转变干部作风、加强机关行政效能建设活动，实现工作作风明显改进，服务能力明显增强，办事效率明显提高，发展环境明显优化。

　　推进政府管理创新。切实转变政府职能，推进政企、政资、政事分开，履行经济调节、社会管理、公共服务和市场监管职能，着力建设服务型政府。认真贯彻人大监督法，自觉接受各方面监督。创新政府管理方式，进一步推行政务公开，加快电子政务建设，增强政府工作的透明度，提高政府的公信力和执行力。

　　加强廉政建设。增强忧患意识、公仆意识、节俭意识，身体力行胡锦涛同志倡导的八个方面良好作风，坚定不移地抓好党风廉政建设和反腐败工作，建立健全教育、制度、监督并重的惩治和预防腐败体系，规范政府行为，强化对权力运行的监督，坚决纠正损害群众利益的不正之风，廉洁从政。

　　各位代表！

新的起点，就是新的希望；新的目标，就是新的责任。让我们在市委的正确领导下，振奋精神、坚定信心，锐意进取、励精图治，全面完成今年各项目标任务，为实现“三个率先”，建设富裕和谐防城港而奋斗!
